# Responses of the two‐spotted oak buprestid, Agrilus biguttatus (Coleoptera: Buprestidae), to host tree volatiles

**DOI:** 10.1002/ps.4208

**Published:** 2016-01-25

**Authors:** József Vuts, Christine M Woodcock, Mary E Sumner, John C Caulfield, Katy Reed, Daegan JG Inward, Simon R Leather, John A Pickett, Michael A Birkett, Sandra Denman

**Affiliations:** ^1^ Department of Biological Chemistry and Crop ProtectionRothamsted ResearchHarpendenHertsUK; ^2^ Centre for Ecosystems, Society and Biosecurity, Forest ResearchUK; ^3^ Department of Crop and Environment SciencesHarper Adams UniversityUK

**Keywords:** Agrilus biguttatus, attractant, tree volatiles, acute oak decline, forestry, semiochemical, Buprestidae

## Abstract

**BACKGROUND:**

Agrilus bigutattus (Fabricius) is a forest pest of increasing importance in the United Kingdom. The larvae damage weakened native oaks and are thought to contribute to premature tree death. Suspected links with acute oak decline (AOD) are not yet confirmed, but AOD‐predisposed trees appear to become more susceptible to A. biguttatus attack. Thus, management may be necessary for control of this insect. To explore the possibility of monitoring beetle populations by baited traps, the host tree volatiles regulating A. biguttatus–oak interactions were studied.

**RESULTS:**

Biologically active volatile organic compounds in dynamic headspace extracts of oak foliage and bark were identified initially by coupled gas chromatography–electroantennography (GC‐EAG) and GC–mass spectrometry (GC‐MS), and the structures were confirmed by GC coinjection with authentic compounds. Of two synthetic blends of these compounds comprising the active leaf volatiles, the simpler one containing three components evoked strongly positive behavioural responses in four‐arm olfactometer tests with virgin females and males, although fresh leaf material was more efficient than the blend. The other blend, comprising a five‐component mixture made up of bark volatiles, proved to be as behaviourally active for gravid females as bark tissue.

**CONCLUSIONS:**

These initial results on A. biguttatus chemical ecology reveal aspects of the role of attractive tree volatiles in the host‐finding of beetles and underpin the development of semiochemically based surveillance strategies for this forest insect. © 2015 The Authors. *Pest Management Science* published by John Wiley & Sons Ltd on behalf of Society of Chemical Industry.

## 1 INTRODUCTION

The two‐spotted oak buprestid, Agrilus biguttatus (Fabricius) (Coleoptera: Buprestidae), causes damage to native oak trees in Europe, and is an exotic organism of high invasive risk to the United States.^1^ In Europe, incidences of damage have become more frequent during the last two decades,^2^, ^3^ while specifically for the United Kingdom, trees colonised by A. biguttatus show symptoms of acute oak decline (AOD), a distinctive novel form of oak decline increasingly reported on native British oak species, Quercus petraea [(Mattuschka) Leibl.] and Q. robur (L.) (Fagaceae).^4^ Notably, AOD distribution in the United Kingdom currently covers a similar range to that of A. biguttatus, and a 95% co‐occurrence of symptomatic trees with larval galleries has been reported.^4^, ^5^ The relationship between AOD and A. biguttatus is, however, as yet unclear. The beetle may vector AOD necrosis bacteria, serving as a means of introducing them into trees, thus being essential for AOD development,^4^ may merely be a secondary coloniser of trees already infected by bacteria or just a coincidental visitor.^5^ AOD often leads to tree mortality within just a few years of the appearance of the first symptoms, i.e. cracks between bark plates from which dark fluid seeps and inner bark necrosis due to bacterial activity,^4^ about 5.3% of symptomatic trees having died in monitored sites over 3–4 years of assessment^5^ (Brown N et al., unpublished).

Owing to the unacceptable levels of loss and premature death of high‐value timber trees in the United Kingdom, there is a need to consider possible management options, including highly specific, targeted trapping of A. biguttatus 5 in areas where important timber trees are grown and losses to AOD are high. Management of the pest via this route can potentially be achieved via the deployment of semiochemicals (naturally occurring behaviour‐ and development‐modifying chemicals), such as plant and bacterial volatiles or pheromones, that act by a non‐toxic mode of action and that are as yet unknown for A. biguttatus. Semiochemicals have already proved to be effective in the early detection and monitoring of a wide range of agricultural and forest pests, including the closely related emerald ash borer, Agrilus planipennis Fairmaire, in North America.^6^, ^7^ As part of their life cycle, adult A. biguttatus beetles emerging from trunks in May–July move up to the canopy of mature oak trees to feed on the foliage and mate, followed by gravid females moving down to the trunks to lay their eggs in bark crevices.^5^, ^8^ Visual cues may play a role in these behaviours as a consequence of the colour preference of this species.^5^, ^9^ However, it is likely that attractive leaf and bark volatiles are also important in the behavioural ecology of this pest. To test the hypothesis that (i) virgin females and males of A. biguttatus utilise foliar volatile cues to find feeding and mating sites, and that (ii) gravid females are guided by bark volatiles to oviposition sites, we studied the behavioural responses of adult beetles to oak odour in laboratory olfactometer bioassays, captured volatiles from the leaves and bark by dynamic headspace collection and subjected volatile extracts to antennal electrophysiology to locate active compounds in gas chromatography. Finally, we constructed synthetic blends of identified bioactive compounds and assessed them in behavioural assays.

## 2 MATERIALS AND METHODS

### 2.1 Insect material

Agrilus biguttatus laboratory cultures were established by collecting Q. robur logs from infested trees at different sites across England south of the Midlands (Norfolk–Derbyshire line), from which planks of the bark + sapwood were cut for ease of handling and placed in emergence cages in a greenhouse at ambient temperatures. Emerging adults were sexed and transferred into plastic screw‐top jars (diameter 12 cm, height 19.5 cm) with the bottom removed and a piece of fine mesh held over the top with rubber bands. Beetles were fed fresh oak leaves and were used as required.

### 2.2 Preparation of oak volatile extracts by dynamic headspace collection (air entrainment)

Volatile sampling (Pye volatile collection kit, Kings Walden, Herts, UK) from foliage of live non‐AOD‐symptomatic Q. robur trees was done at Richmond Park, London (lat./long. 51.447453, −0.293134), in July 2013. A bunch of 20 leaves, virtually free from pest and disease damage, at a canopy height of 2 m was enclosed in a transparent cooking bag (Sainsbury's Supermarkets Ltd, UK). Air was pumped (12 V DC pump; KNF, Reiden, Switzerland) through an activated charcoal filter at 600 mL min^−1^ into each bag, using Teflon tubing, to provide a positive pressure of clean air.^10^ One of the top corners of the cooking bag was snipped off to make an opening, into which a glass tube (8 cm × 0.3 cm i.d.) containing 50 mg of Porapak Q adsorbent sandwiched between glass wool plugs was inserted, and the bag was sealed with wire ties. Air was drawn from the bag through the tube under negative pressure at a flow rate of 500 mL min^−1^. In this way, volatile compounds were collected on Porapak Q traps for 8 h during daylight and were then eluted from the adsorbent with 750 µL of freshly distilled diethyl ether. Extracts were concentrated to 100 µL under a gentle nitrogen stream and kept at −20 °C until use. Volatiles from oak bark planks (∼30 × 20 × 6 cm), removed from felled non‐AOD‐symptomatic Q. robur trees, were collected in a similar way by placing the planks in glass containers (50 × 30 × 20 cm), each with a metal lid, to which the air inlet and outlet were attached. Samplings were run in the lab for 72 h.

### 2.3 Analysis of oak volatile extracts by coupled gas chromatography–electroantennography (GC‐EAG)

To locate bioactive compounds, air entrainment samples from oak leaves were tested against virgin A. biguttatus female and male antennae, and those prepared from oak logs were tested against gravid female antennae. The GC‐EAG system has been described previously.^11^ Antennal recordings (n = 5 each for virgin females, males and gravid females) were made using Ag–AgCl glass electrodes filled with saline solution. An antenna was excised and suspended between the two electrodes. The tip of the terminal process of the antenna was removed to ensure a good contact. Aliquots of the concentrated air entrainment extracts (1 µL) were injected into the GC. The signals were passed through a high‐impedance amplifier (UN‐06; Syntech, Kirchzarten, Germany). Separation of the volatiles was achieved on a GC (6890 N; Agilent Technologies, Santa Clara, CA) equipped with a cool on‐column injector and an FID, using a 50 m × 0.32 mm i.d. HP‐1 column. The oven temperature was maintained at 30 °C for 2 min and then programmed at 15 °C min^−1^ to 250 °C. The carrier gas was helium. The outputs from the EAG amplifier and the FID were monitored simultaneously and analysed using a customised software package (Syntech). A compound was defined as EAG active if it evoked an antennal response, distinguishable from background noise, in three or more coupled runs.

### 2.4 Analysis of volatiles by coupled GC–mass spectrometry (GC‐MS)

For the identification of electrophysiologically active compounds in air entrainment samples, an Agilent 6890 N GC fitted with a capillary GC column (50 m × 0.32 mm i.d. HP‐1, 0.52 µm film thickness; J&W Scientific, Folsom, CA) and equipped with a cool on‐column injector was directly coupled to a mass spectrometer (Micromass Autospec Ultima; Waters, Milford, MA). Ionisation was by electron impact at 70 eV, 220 °C. The oven temperature was maintained at 30 °C for 5 min and then programmed at 5 °C min^−1^ to 250 °C (hold time 21 min). Tentative identification by GC‐MS was confirmed by comparing retention indices of peaks with those of synthetic standards and by peak enhancement on GC by coinjection with authentic compounds,^12^ using an Agilent 6890A GC equipped with a cool on‐column injector, FID and a 50 m × 0.32 mm i.d. HP‐1 column (0.52 µm film thickness). The oven temperature was maintained at 30 °C for 2 min and then programmed at 10 °C min^−1^ to 250 °C. The carrier gas was helium. Quantification of compounds was achieved using known amounts of a series of C7‐C22 alkanes as external standards, although it is appreciated that this results in very small discrepancies.

### 2.5 Chemicals

Authentic samples of the compounds tentatively identified by GC‐MS were purchased for structure confirmation by GC peak enhancement and for behavioural assays. (Z)‐3‐Hexenal (50% solution in triacetin), (E)‐2‐hexenal (99%), p‐cymene (99%), 1,8‐cineole (99%), γ‐terpinene (95%), linalool oxide [2‐(5‐methyl‐5‐vinyltetrahydro‐1‐furyl)‐2‐propanol] (>97%) and (R/S)‐camphor (96%) were obtained from Sigma‐Aldrich (Gillingham, Dorset, UK). (Z)‐3‐Hexen‐1‐ol (98%) and (Z)‐3‐hexenyl acetate (99%) were purchased from Alfa Aesar (Heysham, Lancs, UK), and (Z)‐ocimene (90%) was obtained from Bush Boake Allen (London). 3‐Ethylacetophenone (95%) was obtained from Maybridge (Tintagel, Cornwall, UK). (E)‐Ocimene (95%) was synthesised according to Chou et al. 13 (E)‐4,8‐Dimethyl‐1,3,7‐nonatriene (95.6%) was synthesised from geraniol by oxidation to the corresponding aldehyde, followed by Wittig methylenation.^14^

### 2.6 Assessment of beetle behaviour in olfactometer bioassays

To determine beetle behavioural responses, we used a Perspex four‐arm olfactometer.^15^, ^16^ The olfactometer consisted of three layers of Perspex, held together with plastic nuts and bolts. Both the top and bottom discs had a 156 mm diameter and 5 mm thickness, and the bottom disc was fitted with a filter paper base to provide traction for the walking insect. The middle part was 180 mm in diameter and 7 mm thick and was manufactured to embody four side areas or arms (55 mm in length × 5 mm height each) situated at 90° to each other. The areas narrowed towards the perimeter and were connected to glass chambers, holding plant material, with Teflon tubing, or to glass arms (narrow part: 50 mm in length × 2.5 mm in diameter; wide part: 90 mm in length × 20 mm in diameter) through a 3 mm diameter hole at the end of each of the four arms. Prior to each experiment, all glassware was washed with Teepol detergent, rinsed with acetone and distilled water and baked in an oven overnight at 130 °C. Perspex components were washed with Teepol solution, rinsed with 80% ethanol solution and distilled water and left to air dry. The olfactometer was illuminated from above by diffuse uniform lighting from two 18 W/35 white fluorescent light bulbs. It was surrounded by black paper to remove any external visual stimuli. A single beetle was introduced into the olfactometer at each test period. Air was drawn through the central hole by a vacuum pump (220–240 V AC; Charles Austen Pumps Ltd, Byfleet, Surrey, UK) and thereby pulled through each of the four side arms (75 mL min^−1^ arm^−1^) and subsequently exhausted from the room. Each beetle was given 2 min to acclimatise, after which the experiment was run for 16 min at 24 ° C, the olfactometer being rotated by 90 deg every 4 min to control for any directional bias. The olfactometer was divided into four regions corresponding to each of the four arms, and the time spent in each arm by a single beetle was recorded using specialist software (OLFA, Udine, Italy). Data were analysed statistically by ANOVA at α = 0.05, followed by Fisher's LSD test (GenStat 11th edition; VSN International Ltd, Hemel Hempstead, Herts, UK).

Based on the expectation that, similarly to other insect species,^17^, ^18^ mating alters A. biguttatus olfactory preference from feeding to oviposition site‐related odours, we used virgin females and males to assess behavioural responses to oak foliage. Foliage material (twig with 20 leaves) was put in a closed glass vessel (19 × 10 cm diameter) connected with Teflon tubing to one of the side arms (test 1). For tests with oak bark (test 6; 20 × 6 × 3 cm piece in vessel), gravid females were used. Empty glass vessels served as the three controls. This set‐up increases the statistical power of the experiment by making it less likely for an insect accidentally to wander into/out of the treated region.^15^

To assess the activity of synthetic blends, the test compounds were administered in 10 µL of hexane on a ca 2 cm^2^ piece of filter paper (Whatman™, Maidstone, Kent, UK). After the solvent evaporated (30 s), it was placed in a glass arm (test arm). The same amount of hexane solvent was used in each of the three control glass arms (10 µL on filter paper). Two synthetic blends, comprising volatiles identified from leaves, were constructed (tests 2 to 5). Blend 1 comprised all the EAG‐active leaf volatiles in the same ratios as found in GC/GC‐MS analyses. The amounts of compounds used in each test were similar to those released by ca 20 leaves in 8 h: (Z)‐3‐hexenal 0.04 µg, (E)‐2‐hexenal 0.12 µg, (Z)‐3‐hexen‐1‐ol 1 µg, (Z)‐3‐hexenyl acetate 0.33 µg, (Z)‐ocimene 0.25 µg, (E)‐ocimene 1.1 µg, linalool oxide 0.12 µg, (E)‐4,8‐dimethyl‐1,3,7‐nonatriene (DMNT) 0.8 µg and m‐ethylacetophenone 0.16 µg. Blend 2 contained (Z)‐3‐hexenal, (Z)‐3‐hexen‐1‐ol and (Z)‐3‐hexenyl acetate in the same proportions as in blend 1. The rationale for blend 2 was that, in a preliminary study (Vuts J et al., unpublished), antennae of A. biguttatus responded strongly to the above three components of an air entrainment extract of oak leaves (see supporting information Fig. S1). The synthetic blend of electrophysiologically active bark volatiles (blend 3) comprised p‐cymene 0.29 µg, 1,8‐cineole 0.54 µg, (E)‐ocimene 1 µg, γ‐terpinene 0.26 µg and (R/S)‐camphor 0.24 µg. The amount of each compound used was calculated to be similar to that collected from the bark used in the bioassays.

## 3 RESULTS

### 3.1 Identification of electrophysiologically active compounds in oak foliage and bark headspace extracts

**Figure 1:**
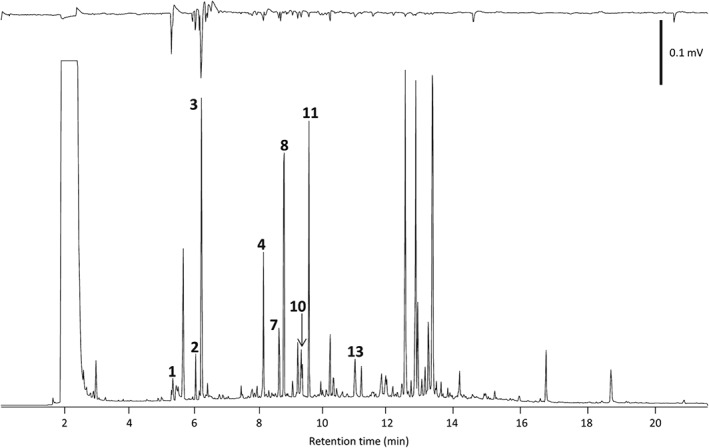
GC‐EAG profile of an oak foliage air entrainment extract tested on a virgin male Agrilus biguttatus antenna. Upper trace: EAG response; lower trace: GC FID response. The numbers refer to compounds that elicited EAG responses from three or more antennae: **1** (Z)‐3‐hexenal; **2** (E)‐2‐hexenal; **3** (Z)‐3‐hexen‐1‐ol; **4** (Z)‐3‐hexenyl acetate; **7** (Z)‐ocimene; **8** (E)‐ocimene; **10** linalool oxide (for isomer, see Section 2); **11** (3E)‐4,8‐dimethyl‐1,3,7‐nonatriene; **13** m‐ethylacetophenone. For numbering, see Table 1

**Figure 2:**
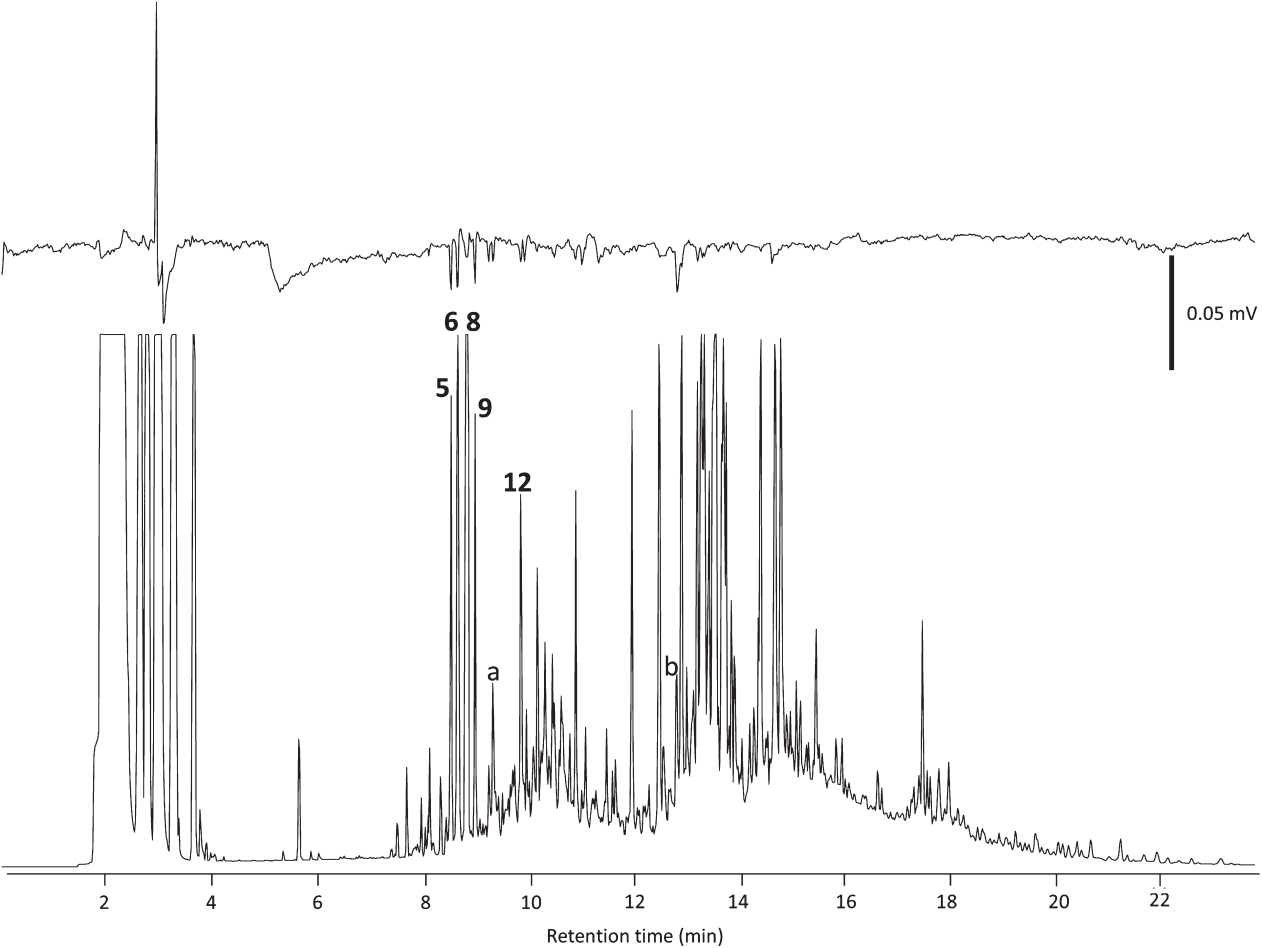
GC‐EAG profile of an oak bark air entrainment extract tested on a gravid female Agrilus biguttatus antenna. Upper trace: EAG response; lower trace: GC FID response. The numbers refer to compounds that elicited EAG responses from three or more antennae: **5** p‐cymene; **6** 1,8‐cineole; **8** (E)‐ocimene; **9** γ‐terpinene; **12** camphor. For numbering, see Table 1. Other, tentatively identified, compounds: a nonanal; b caryophyllene

Coupled GC–electrophysiology (GC‐EAG), using the antennae of virgin female and male A. biguttatus, as well as mated female A. biguttatus, with the volatiles collected from oak foliage and bark respectively, revealed the presence of several EAG‐active compounds, which were identified by coupled GC‐MS and GC peak enhancement (Table 1). For virgin females and males, EAG‐active compounds were identified as (Z)‐3‐hexenal, (E)‐2‐hexenal, (Z)‐3‐hexen‐1‐ol, (Z)‐3‐hexenyl acetate, (Z)‐ocimene, (E)‐ocimene, linalool oxide (for isomer, see Section 2), (E)‐4,8‐dimethyl‐1,3,7‐nonatriene (DMNT) and 3‐ethylacetophenone (Fig. 1). For mated females, active compounds were identified as p‐cymene, 1,8‐cineole, (E)‐ocimene, γ‐terpinene and camphor (Fig. 2). The stereochemistry of the camphor was not determined.

**Table 1:** Electrophysiologically active compounds identified in volatile samples of oak (Quercus robur) foliage (a) and bark (b) for virgin female, male and gravid female Agrilus biguttatus. Identifications were made by GC‐MS and confirmed by GC peak enhancement studies (see Section 2). Quantifications were made using known amounts of external standards (a series of C7–C22 alkanes)

Number	Compound	Retention index^a^ (non‐polar)	Concentration (ng µL^−1^)
**1**	(Z)‐3‐Hexenal (a)	800	3.1
**2**	(E)‐2‐Hexenal (a)	825	9.1
**3**	(Z)‐3‐Hexenol (a)	841	78.4
**4**	(Z)‐3‐Hexenyl acetate (a)	988	25.0
**5**	p‐Cymene (b)	1017	25.8
**6**	1,8‐Cineole (b)	1022	49.7
**7**	(Z)‐Ocimene (a)	1028	20.2
**8**	(E)‐Ocimene (a, b)	1041	80.1 (a), 84.8 (b)
**9**	γ‐Terpinene (b)	1058	24.6
**10**	Linalool oxide (a) (for isomer, see Section 2)	1063	9.2
**11**	(E)‐4,8‐Dimethyl‐1,3,7‐nonatriene (a)	1106	63.2
**12**	(R/S)‐Camphor (b)	1134	23.4
**13**	m‐Ethylacetophenone (a)	1250	11.7

a On an HP‐1 GC column (Hewlett‐Packard, Palo Alto, CA).

### 3.2 Assessment of beetle behaviour in olfactometer bioassays

Virgin female and male *A. biguttatus* spent more time in the arm of the olfactometer containing odour from oak leaf material than in control arms (test 1) (Table 2). Using two synthetic blends of EAG‐active compounds from oak foliage [blend 1 = (*Z*)‐3‐hexenal, (*E*)‐2‐hexenal, (*Z*)‐3‐hexen‐1‐ol, (*Z*)‐3‐hexenyl acetate, (*Z*)‐ocimene, (*E*)‐ocimene, linalool oxide (for isomer, see Section 2), DMNT, *m*‐ethylacetophenone; blend 2 = (*Z*)‐3‐hexenal, (*Z*)‐3‐hexen‐1‐ol, (*Z*)‐3‐hexenyl acetate], females and males spent more time in the arm containing either of the synthetic leaf blends than in the control arms (tests 2 and 3) (Table 2). When blends 1 and 2 were compared directly, virgin females showed no significant preference for either blend; however, male beetles spent a longer time in the arm containing blend 2 than in the arm containing blend 1 (*P* = 0.026; test 4) (Table 2). When oak foliage and blend 2 were compared directly, no significant difference was observed for either sex, but males preferred blend 2 to the control (*P* < 0.001 for both sexes; test 5) (Table 2). Gravid females spent more time in the arm containing odour from oak bark than in the control arms (test 6) (Table 2), and a synthetic blend of EAG‐active compounds isolated and identified from bark [blend 3 = *p*‐cymene, 1,8‐cineole, (*E*)‐ocimene, *γ*‐terpinene, (*R*/*S*)‐camphor] was significantly more active than the control solvent (test 7) (Table 2). There was no significant difference in the response of females to oak bark and blend 3, with both being more active than the control (*P* < 0.017; test 8) (Table 2).

**Table 2:** Behavioural response of virgin female, male and gravid female Agrilus biguttatus to the odour of oak foliage and bark and synthetic blends of identified electrophysiologically active compounds (see Table 1 for list of compounds). Response was measured as the mean (± SE) time spent in the arms of the olfactometer. The control comprised empty glass vessels in tests 1 and 6, and hexane in tests 2 to 5 and 7 to 8. Treatments with the same letter do not differ significantly from each other (ANOVA, α = 0.05; P‐values from Fisher's LSD test). n.t.: not tested

Test number	Treatments	Virgin female			Male		
		Mean time spent (min) ± SE	*P*	Number of replicates	Mean time spent (min) ± SE	*P*	Number of replicates
1	Oak foliage	4.42 ± 0.52	<0.001	10	3.81 ± 0.26	<0.001	10
	Control	1.09 ± 0.3			1.27 ± 0.15		
2	Blend 1	2.66 ± 0.45	0.048	10	3.77 ± 0.88	0.013	10
	Control	1.57 ± 0.26			1.86 ± 0.72		
3	Blend 2	2.45 ± 0.37	0.029	11	4.73 ± 1.32	0.037	10
	Control	1.48 ± 0.21			2.36 ± 1.08		
4	Blend 1	1.6 ± 0.32	AB	22	2.22 ± 1.17	a	10
	Blend 2	1.84 ± 0.32	B		4.42 ± 1.17	b	
	Control	1.2 ± 0.23	A		2.18 ± 0.96	a	
5	Oak foliage	2.88 ± 0.29	B	10	2.22 ± 0.27	c	14
	Blend 2	0.94 ± 0.21	A		1.34 ± 0.16	b	
	Control	0.87 ± 0.09	A		0.95 ± 0.12	a	
**Test number**	**Treatments**	**Gravid female**			**Male**		
		**Mean time spent (min) ± SE **	***P***	**Number of replicates**	**Mean time spent (min) ± SE**	***P***	**Number of replicates**
6	Oak bark	3.02 ± 0.52	<0.001	10	n.t.		
	Control	0.67 ± 0.3					
7	Blend 3	2.49 ± 0.43	0.025	12	n.t.		
	Control	1.31 ± 0.25					
8	Oak bark	1.78 ± 0.43	B	10	n.t.		
	Blend 3	1.57 ± 0.25	B				
	Control	0.79 ± 0.13	A				

## 4 DISCUSSION

In this study it has been demonstrated that, under laboratory conditions, virgin *A. biguttatus* individuals show odour‐guided orientation towards volatile organic compounds emitted from fresh oak foliage. This supports the hypothesis that *A. biguttatus* uses olfactory signals to locate their host trees. Furthermore, *A. biguttatus* antennae respond to specific components of oak leaf odour, suggesting their involvement in host location. Responses to green leaf volatiles (*Z*)‐3‐hexenal, (*Z*)‐3‐hexen‐1‐ol, (*E*)‐2‐hexenal and (*Z*)*‐*3‐hexenyl acetate were observed, similarly to *A. planipennis*, where a suite of green leaf volatiles from ash foliage evoked strong activity.^19^, ^20^ Interestingly, the amplitudes of EAG responses of *A. planipennis* to (*Z*)‐3‐hexenal, (*Z*)‐3‐hexen‐1‐ol and (*Z*)*‐*3‐hexenyl acetate observed by de Groot *et al.* 20 show a similar pattern to that observed in the present study with *A. biguttatus*, (*Z*)‐3‐hexen‐1‐ol eliciting a greater electrical signal than either (*Z*)‐3‐hexenal or (*Z*)*‐*3‐hexenyl acetate. Furthermore, *A. biguttatus* males gave generally stronger EAG responses than females, a phenomenon also described in *A. planipennis*.^20^ It should be noted, however, that EAG responses are regarded as qualitative rather than quantitative indicators of a compound's activity.^21^ In subsequent behavioural experiments, synthetic blends comprising the EAG‐active compounds evoked positive responses for both sexes. Interestingly, the reduced blend containing only three of the nine EAG‐active volatiles was more active than the complete blend. Although we cannot yet explain this finding, it may be that the synthetic blend of all EAG‐active compounds contains some that contribute inhibitor activity, thereby reducing the overall activity of the mixture, or it may be repellent. The behavioural role of a compound cannot be predicted on the basis of its EAG activity,^11^ only a series of bioassays can establish that conclusively. In addition, a growing body of evidence suggests that odour perception is synthetic, i.e. the individual components of a mixture may no longer be recognisable in the blend and the mixture is perceived as a distinct odour different from its individual components.^22^ The aphid *Aphis fabae* (Scop.) (Hemiptera: Aphididae), for example, was repelled by the individual components of a volatile blend from its host plant; however, when the blend itself was presented to the aphids, a positive response was elicited.^23^ Other EAG‐active oak foliage compounds, DMNT and (*Z*)‐ and (*E*)‐ocimene, are plant stress compounds related to insect herbivory.^24^, ^25^ It would be interesting to compare their emission from damaged and undamaged oak foliage, as beetle damage may induce the production of stress volatiles, which then induce the aggregation of *A. biguttatus* at the damage site. Linalool oxide is a widespread plant compound occurring in flowers, fruit and vegetative parts,^26^ whereas m‐ethylacetophenone has only been identified from the floral bouquet of an orchid.^27^

Five bark volatile compounds produced stable antennal responses on the antennae of gravid females, a synthetic blend of which elicited as strong a positive orientation response from females as the bark tissue. Gravid *A. planipennis* females were found to give stronger EAG responses to ash bark volatiles than virgin females,^28^ suggesting that mating induces physiological changes in the peripheral olfactory apparatus. This might also be the case for *A. biguttatus*, but a systematic study is needed involving virgin beetles. Essential oils from *Phoebe porosa* (Mez.) (Lauraceae) (phoebe oil) and *Leptospermum scoparium* (Forst. and Forst.) (Myrtaceae) (manuka oil), containing all or a subset of the EAG‐active ash bark volatiles, proved to be highly active in the field, resulting in high trap catches of *A. planipennis*.^29^ *p*‐Cymene, 1,8‐cineole, *γ*‐terpinene and (*R*/*S*)‐camphor in the present study are widespread volatiles identified from different plant parts, including bark, across a range of taxa.^26^, ^30^, ^31^, ^32^ (*E*)‐Ocimene was also found in the headspace of tree bark by studies elsewhere.^33^, ^34^

From a chemical ecology perspective, *A. planipennis* is the most thoroughly studied buprestid species^28^, ^35^ owing to its pest status in North America.^36^ The host volatile, (*Z*)‐3‐hexen‐1‐ol, identified from ash foliage, is highly attractive to males and, to a lesser extent, to females.^20^ In our study, at the behavioural level, *A. biguttatus* males exhibited a higher degree of olfactory discrimination between the synthetic blends than females. The function of this might be similar to a behaviour reported from *A. planipennis*, where females call most frequently on host foliage, and hence the host volatile, (*Z*)‐3‐hexen‐1‐ol, synergises the attraction of males to the female‐produced sex pheromone.^7^ A similar scenario could be possible in *A. biguttatus*. Nothing, however, is known about its pheromone communication, and this requires further investigation.

The chemical ecology of another oak tree/buprestid relationship was recently investigated between the cork oak (*Quercus suber* L.) and its pest, *Coroebus florentinus* Herbst (Agrilinae). Antennae of both sexes responded to the green leaf volatiles (*E*)‐2‐hexenal, (*E*)‐2‐hexenol, 1‐hexanol, (*Z*)‐3‐hexenyl acetate and *n*‐hexyl acetate, all identified from the freshly cut leaf‐bearing branches of the host plant.^37^ In contrast to our studies with *A. biguttatus*, in laboratory behavioural experiments, only females showed a positive response to headspace samples from the host foliage, as well as to a synthetic blend of the constituents (*E*)‐2‐hexenol, 1‐hexanol and (*Z*)‐3‐hexenyl acetate. Fürstenau *et al.* 37 propose a role for these green leaf volatiles in foraging and/or oviposition behaviour of *C. florentinus*.

Other investigations have focused on the field testing of synthetic leaf volatile compounds that had been described from other tree–insect relationships. For example, females of the cork oak pest *C. undatus* Fabricius were attracted to a synthetic mixture of green leaf volatiles [(*E*)‐2‐hexenal, (*E*)‐2‐hexenol, 1‐hexanol, (*Z*)‐3‐hexenyl acetate and n‐hexyl acetate] identified from *Q. suber* foliage.^38^ Coleman *et al.* 39 investigated the field responses of *Agrilus auroguttatus* Schaeffer, a pest of the native Californian oak species *Quercus agrifolia* Née, to three different semiochemical lures, namely (*Z*)‐3‐hexenol, manuka oil and phoebe oil. They found that the addition of any of the commercially available lures significantly enhanced male, but not female, trap catch when compared with unbaited control traps, and that there were no differences in trap catch between the lures. Some species of the *Buprestis*, *Chalcophora* and *Chrysobothris* genera are attracted to chemical compounds such as ethanol and various monoterpenes (*α*‐pinene and 3‐carene), which are released by trees under abiotic or biotic stress.^40^, ^41^, ^42^

Our results provide the first evidence that *A. biguttatus* responds to volatiles from its oak tree host. Field trials with synthetic blends of identified compounds are planned to optimise lures for use in early detection and monitoring programmes. Much is still to be learned about the semiochemicals governing interactions between *A. biguttatus* and oak trees, including the effect of the AOD infection stage on leaf volatile emissions, as well as the role that AOD‐associated bacteria and different fungi play in the attraction of gravid females to tree trunks. Also, research into the pheromone biology of *A. biguttatus* may facilitate new discoveries enabling study of possible synergistic interactions between plant volatiles and pheromones, leading to enhanced surveillance of this forest insect.

## Supporting information

FigureS1
