# Supplementary figures and images for: Responses of the two‐spotted oak buprestid, Agrilus biguttatus (Coleoptera: Buprestidae), to host tree volatiles
Source: Pest Manag Sci. 2016 Jan 25;72(4):845–51. doi: 10.1002/ps.4208 (PMC5066750; doi:10.1002/ps.4208)

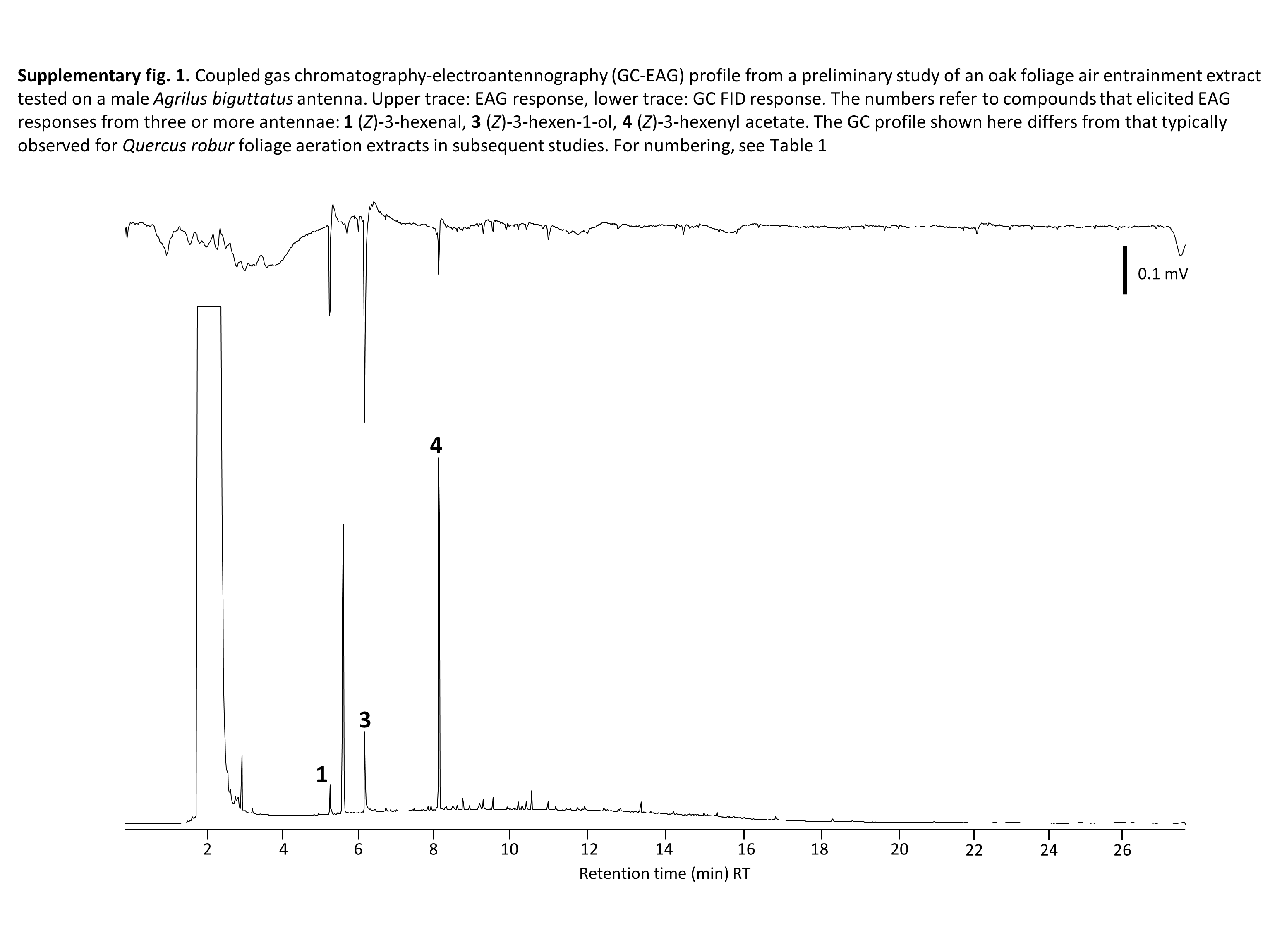

Supplement: Supplementary file 1 — FigureS1 [file PS-72-845-s001.tif]
